# Elevated Plasma Vitamin B12 Concentrations Are Independent Predictors of In-Hospital Mortality in Adult Patients at Nutritional Risk

**DOI:** 10.3390/nu9010001

**Published:** 2016-12-23

**Authors:** Silvia Cappello, Emanuele Cereda, Mariangela Rondanelli, Catherine Klersy, Barbara Cameletti, Riccardo Albertini, Daniela Magno, Marilisa Caraccia, Annalisa Turri, Riccardo Caccialanza

**Affiliations:** 1 Nutrition and Dietetics Service, Fondazione IRCCS Policlinico San Matteo, Pavia 27100, Italy; cappellosilvia@hotmail.it (S.C.); e.cereda@smatteo.pv.it (E.C.); barbara_cameletti@inwind.it (B.C.); daniela.magno@gmail.com (D.M.); marilisacaraccia@virgilio.it (M.C.); turriannalisa1@gmail.com (A.T.); 2 Experimental and Forensic Medicine, Department of Public Health, University of Pavia, Section of Human Nutrition, Endocrinology and Nutrition Unit, Azienda di Servizi alla Persona, Pavia 27100, Italy; mariangela.rondanelli@unipv.it; 3 Biometry and Clinical Epidemiology Service, Fondazione IRCCS Policlinico San Matteo, Pavia 27100, Italy; klersy@smatteo.pv.it; 4 Laboratory Analysis Service, Fondazione IRCCS Policlinico San Matteo, Pavia 27100, Italy; r.albertini@smatteo.pv.it

**Keywords:** vitamin B12, in-hospital mortality, length of stay, nutritional risk

## Abstract

*Background:* Elevated plasma vitamin B12 concentrations were identified as predictors of mortality in patients with oncologic, hepatic and renal diseases, and in elderly and critically ill medical patients. The association between vitamin B12 concentrations and in-hospital mortality in adult patients at nutritional risk has not been assessed. *Methods:* In this five-year prospective study, we investigated whether high vitamin B12 concentrations (>1000 pg/mL) are associated with in-hospital mortality in 1373 not-bed-ridden adult patients at nutritional risk (Nutrition Risk Index <97.5), admitted to medical and surgical departments. *Results:* Three hundred and ninety-six (28.8%) patients presented vitamin B12 > 1000 pg/mL. Two hundred and four patients died in the hospital (14.9%). The adjusted odds ratio of in-hospital mortality in patients with high vitamin B12 was 2.20 (95% CI, 1.56–3.08; *p* < 0.001); it was independent of age, gender, body mass index, six-month previous unintentional weight loss, admission ward, presence of malignancy, renal function, C-reactive protein and prealbumin. Patients with high vitamin B12 also had a longer length of stay (LOS) than those with normal concentrations (median 25 days, (IQR 15–41) versus 23 days (IQR 14–36); *p* = 0.014), and elevated vitamin B12 was an independent predictor of LOS (*p* = 0.027). *Conclusions:* An independent association between elevated vitamin B12 concentrations, mortality and LOS was found in our sample of hospitalized adult patients at nutritional risk. Although the underlying mechanisms are still unknown and any cause-effect relation cannot be inferred, clinicians should be aware of the potential negative impact of high vitamin B12 concentrations in hospitalized patients at nutritional risk and avoid inappropriate vitamin supplementation.

## 1. Introduction

Vitamin B12 deficiency is frequently observed in hospitalized patients [1,2] and is associated with conditions such as anemia and neuropsychiatric disorders [3]. Despite the lack of definitive and clear clinical evidence, vitamin B12 supplementation is diffuse, in particular among elderly patients at nutritional risk, as it is believed to favor strength and appetite recovery and it is known to have some positive effects on mood and neurological functions [4,5], and to protect against cancer [6] and heart disease [7] as well, due to its antioxidant properties [8].

On the other hand, elevated plasma vitamin B12 concentrations have been found to be associated with cancer [9], disease severity in anorexia nervosa [10], and they have been identified as predictors of mortality in patients with oncologic [11,12,13] and hepatic diseases [14], and in elderly [15,16,17], hemodialyzed [18] and critically ill medical patients [19,20]. However, some studies have shown controversial results [21,22,23].

To date, the association between vitamin B12 concentration and in-hospital mortality in adult patients at nutritional risk has not yet been assessed. Hence, in the present prospective observational study, we evaluated the association between elevated vitamin B12 plasma concentrations and in-hospital mortality in a sample of 1373 not-bed-ridden adult patients at nutritional risk with a multivariate approach, which also took into consideration the inflammatory state and renal and hepatic function.

## 2. Methods

### 2.1. Design and Subjects

This prospective hospital-based observational study was carried out in the Italian Research Hospital Fondazione IRCCS Policlinico San Matteo (Pavia, Italy). It was approved (project ID: VitB12, malnutrition and mortality; released on 25 July 2011) by the Ethics Committee of the Fondazione IRCCS Policlinico San Matteo (Pavia, Italy) and performed in agreement with the principles of the Declaration of Helsinki. Over a five-year period (August 2011 to May 2016), adult (≥18 years old) patients admitted to both medical and surgical departments were systematically screened for study inclusion. Obstetrics, intensive care units and other emergency settings were excluded. Recruitment was performed as follows: every four months, for one month, all the subjects newly admitted (within 24 h) were assessed for eligibility. Exclusion criteria were: being bed-ridden, same-day surgery, admitted for a day procedure, palliative care, ongoing artificial nutrition, ongoing or three-month previous vitamin B12 or multivitamins supplementation, which was investigated also checking the general practitioner-provided prescription packages at admission and by careful interviews with relatives or caregivers. All patients at nutritional risk (Nutrition Risk Index < 97.5) potentially recruitable were asked for personal agreement by written informed consent. Nutritional risk was present at admission in 1452 (44%) of the 3300 analyzed patients. Of these, 1373 (94.5%) agreed to participate.

### 2.2. Measurements

Data on height, weight, body mass index (BMI; in kg/m^2^), weight loss and serum albumin were collected within 24 h since admission. When height measurement was inaccurate due to evident deformities of the spinal cord (e.g., kyphosis or scoliosis) stature was calculated from knee height. Nutrition-related risk of complications was assessed by the Nutritional Risk Index (NRI) as previously proposed by Buzby [24]. The NRI is a nutritional score based on serum albumin concentration and the ratio of actual to usual weight (defined as the stable weight in the last 6 months). The NRI score was derived as follows:

NRI = (1.519 × serum albumin, g/L) + (41.7 × present/usual weight)

A NRI score of >100 indicates that patient has no risk; 97.5 to 100, mild risk; 83.5 to 97.5, moderate risk; and <83.5, severe risk. Clinically relevant nutritional risk was defined by a NRI < 97.5 [25,26].

Along with total weight loss data retrieval, patients were also asked to quantify the change in body weight occurred in the six months before admission. A weight loss ≥10% was considered a further indicator of malnutrition.

The following biochemical data were collected within 72 h since admission, after an overnight fast, using serum tubes with clot activators: prealbumin, C-reactive protein (CRP), cholinesterase, creatinine, and vitamin B12. Particularly, total plasma vitamin B12 concentrations were measured with a solid-phase, competitive chemiluminescent enzyme immunoassay analyses on a Immulite^®^ 2000 (Siemens Diagnostic Products Corp., Los Angeles, CA, USA) automated immunochemistry analyzer, which was operated according to manufacturer’s instructions. Reference ranges (cutoffs) were defined as follows: 243–1000 pg/mL.

Glomerular filtration rate was estimated using the Chronic Kidney Disease Epidemiology Collaboration (CKD-EPI) formula [27] and a value <60 mL/min was considered a negative prognostic factor [28].

### 2.3. Study end Points

We investigated whether high vitamin B12 concentrations (>1000 pg/mL) are associated with in-hospital (within 30-day since admission) all-cause mortality. Outcome data were collected through active follow-up or record linkage with regional or national registries. The association between elevated vitamin B12 concentrations and length of stay (LOS) was also assessed.

### 2.4. Sample Size

Data regarding vitamin B12 concentrations and in-hospital mortality do not refer to mixed patient populations [11,12,13,14,15,16,17,18,19,20,21,22]. In a previous study conducted in our institution [25], mortality rate in the sub-group of patients at nutritional risk (NRI < 97.5) was 7%.

Assuming a prevalence of high vitamin B12 of 40% and an estimated mortality of about 8% and 15% in patients with low and high vitamin B12, respectively, we computed that a sample size of 1117 patients would have a power of 80% to detect the hypothesized between-group difference in mortality with a two-sided 0.05 significance level.

### 2.5. Statistical Analysis

All computations were performed with Stata 14.1 (Statacorp, College Station, TX, USA). A two-sided *p*-value < 0.05 was considered statistically significant. Data were described as mean and standard deviation (SD) or median and 25th–75th percentiles if continuous and as counts and percentage if categorical. They were compared between groups of patients with the Student *t* or the Mann Whitney *U* test and the Fisher exact test, respectively.

Mortality rates in patients with low and high vitamin B12 were initially compared using the Fisher’s exact test and the between-group difference with its 95% confidence interval (95% CI) was accordingly computed. Then, the association of mortality and high vitamin B12 was investigated by logistic regression analysis adjusted for potential non collinear confounders. Odds ratios (OR) and 95% CI were computed.

Finally, a multivariable linear regression model was fitted to assess the independent association between elevated vitamin B12 concentrations and LOS (log-transformed).

## 3. Results

A total of 1373 patients met the inclusion criteria. The demographic and clinical characteristics of the study cohort by vitamin B12 concentrations are presented in Table 1. The diagnostic classification (ICD-10) is detailed in Table 2. Overall, 396 (28.8%) patients presented a vitamin B12 value >1000 pg/mL and were characterized by significantly reduced concentrations of albumin, prealbumin and cholinesterase. In 111 patients (8.1%), vitamin B12 was <243 pg/mL; no association with subnormal vitamin B12 concentrations and mortality was detected. Two hundred and four patients died in the hospital (14.9% (95% CI, 13.0–16.9)). The adjusted risk of the in-hospital mortality in patients with high vitamin B12 was 2.20 ((1.56–3.08); *p* < 0.001). In the multivariable analysis the risk of death associated with vitamin B12 > 1000 pg/mL was independent of age, gender, BMI, six-month previous unintentional WL ≥ 10%, admission to a surgical ward, presence of malignant disease, CRP, renal function and prealbumin (Table 3). This significant independent association was also confirmed in those models alternatively including albumin or cholinesterase rather than prealbumin (significantly collinear variables, surrogates of hepatic synthetic function).

Other predictors of death were WL ≥ 10%, kidney failure, CRP ≥ 2.4 mg/dL, prealbumin <10 mg/dL, albumin <3.00 g/dL and cholinesterase <5064 UI/mL.

Patients with a high vitamin B12 level had a longer LOS (median 25 days, IQR 15–41 days) than those with concentrations <1000 pg/mL (median 23 days, IQR 14–36 days) (*p* = 0.014). Elevated vitamin B12 was also found to be a predictor of LOS independent of age, gender, BMI, six-month previous unintentional weight loss, admission ward, presence of malignancy, renal function, C-reactive protein and prealbumin (*p* = 0.027).

## 4. Discussion

In line with the available literature, our research hypothesis was that elevated vitamin B12 concentrations are also associated with higher mortality in adult hospitalized patients at nutritional risk. This was confirmed, as we found a significant association between elevated vitamin B12 concentrations, mortality and LOS in the examined large, mixed clinical population.

The multivariate statistical approach allowed us to detect that this association is independent from other prognostic factors such as renal function, hepatic function, inflammation and the degree of weight loss.

Our results are consistent with previous findings in patients with oncologic [11,12,13] and hepatic diseases [14], and in elderly [15,16,17], hemodialyzed [18] and critically ill medical patients [19,20]. As far as we are concerned, the association between LOS and high vitamin B12 concentrations has not been investigated before.

We chose to focus on patients at nutritional risk, as they represent a particularly prevalent hospital population with the highest mortality rates and LOS [25,26,29].

Patients with or without elevated vitamin B12 concentrations did not differ with regards to the degree of nutritional risk (NRI score, BMI, previous six-month weight loss). However, the 28.8% prevalence of elevated vitamin B12 found represents the highest among those reported by previous studies [30] and might be explained by the presence of impaired nutritional status.

Patients with vitamin B12 values >1000 pg/mL were characterized by significantly reduced concentrations of albumin, prealbumin and cholinesterase, which are all indices of synthetic hepatic function; this evidence is consistent with vitamin B12 metabolism and previous reports in liver diseases [31].

It is known that elevated vitamin B12 plasma concentrations can theoretically be associated with a functional deficiency due to a reduced intracellular concentration as a result of cell damage [31].

The binding of vitamin B12 to haptocorrin can be increased by both a diminished protein clearance by the liver and an elevated production of haptocorrin by an increased number of leukocytes, as in some hematologic disorders, and other cell types. This condition inhibits the binding of vitamin B12 to transcobalamin II, which is the physiologic transport protein required for intracellular uptake, thus leading to elevated vitamin B12 plasma concentrations. They can be also the consequence of a decreased production of transcobalamin II by the liver, which leads to a decreased uptake by peripheral tissues [30,32].

As also suggested in patients affected by anorexia nervosa [10], plasma concentrations of vitamin B12 might be an early marker of liver dysfunction, possibly also related to more severe pathological aspects. The identification of patients with higher fasting plasma vitamin B12 concentrations could therefore lead to earlier and more careful refeeding interventions in patients at nutritional risk. However, this hypothesis must be confirmed by nutritional intervention trials, and the underlying pathophysiological mechanisms which link high vitamin B12 concentrations with increased mortality remain unknown.

Likewise, the independent association between elevated vitamin B12 and LOS appears unexplainable at the moment. Hence, considering the possible presence of a specific functional deficiency, further studies implying the assessment of transcobalamin sub-types are warranted.

We found no differences according to vitamin B12 concentrations in terms of inflammatory state and the presence of oncologic disease. The lack of association between elevated vitamin B12 and C-reactive protein concentrations conflicts with the 2009 study by Corcoran et al. [33], which investigated the relationship between vitamin concentrations and inflammation and found a positive correlation between C-reactive protein and elevated vitamin B12 concentrations in the first two days of admission in intensive care units [33]. Hence, the potential role of B12 status as an acute phase reactant requires further investigation. Our data do not confirm the previous evidence that high vitamin B12 concentrations are associated with the risk of cancer [9], but this discrepancy might also be due to the selection of a population of hospitalized patients at nutritional risk and the close relationship between cancer and nutritional derangements [24,25,29].

Finally, a potential limitation is that, in line with the available literature, we have arbitrarily adopted a value >1000 pg/mL to define elevated vitamin B12 concentrations. However, specific threshold values for clinical practice still need to be defined.

In conclusion, we found an independent association between elevated vitamin B12 concentrations, mortality and LOS in hospitalized patients at nutritional risk. So, beside the potential importance of the vitamin B12 assay as an early diagnostic marker of several diseases, elevated vitamin B12 concentrations might support clinicians in identifying hospitalized patients with possible worse prognoses among those who are malnourished and thus, per se, at higher risk. Although the results should be interpreted with caution, as the underlying mechanisms are still unknown and no cause-effect relation can yet be inferred, clinicians should also be aware of the potential negative impact of high vitamin B12 concentrations in those patients and avoid uncontrolled, inappropriate vitamin supplementation until clinical studies implying the assessment of transcobalamin sub-types will clarify its potential utility or harm. Nonetheless, the identification of more definite threshold values of elevated vitamin B12 for clinical practice is warranted.

## 5. Conclusions

An independent association between elevated vitamin B12 concentrations, mortality and LOS was found in our sample of hospitalized adult patients at nutritional risk. Although the underlying mechanisms are still unknown and any cause-effect relation cannot be inferred, clinicians should be aware of the potential negative impact of high vitamin B12 concentrations in hospitalized patients at nutritional risk and avoid inappropriate vitamin supplementation.

## Figures and Tables

**Table 1:** Baseline characteristics of the study population by vitamin B12 concentrations ≤/>1000 pg/mL.

	Overall Population (*n* = 1373)	Vitamin B12 ≤ 1000 pg/mL (*n* = 977)	Vitamin B12 > 1000 pg/mL (*n* = 396)	*p*-Value *
Age (years), Mean (SD)	66.7 (15.5)	67.0 (16.0)	66.0 (14.1)	66.0 (14.1)
Male gender, *n* (%)	762 (55.5)	532 (54.4)	230 (58.1)	0.23
Surgical setting, *n* (%)	274 (20.2)	197 (19.4)	77 (19.9)	0.43
BMI (kg/m^2^), Mean (SD)	21.4 (4.8)	21.5 (4.9)	21.1 (4.4)	0.20
Cancer diagnosis, *n* (%)	640 (46.7)	443 (45.3)	197 (49.7)	0.07
Six-month WL (kg), Mean (SD)	9.6 (9.5)	9.5 (10.0)	10.0 (9.8)	0.32
Six-month WL ≥ 10%, *n* (%)	623 (45.4)	433 (44.3)	190 (48.0)	0.23
Albumin (g/dL), Mean (SD)	2.83 (0.62)	2.86 (0.61)	2.74 (0.65)	0.001
NRI score, Mean (SD)	72.3 (17.2)	72.7 (17.3)	71.5 (16.8)	0.23
Prealbumin (mg/dL), Mean (SD)	14.2 (7.8)	14.7 (7.8)	12.9 (7.7)	<0.001
Cholinesterase (UI/mL), Mean (SD)	4107 (1871)	4311 (1881)	3684 (1781)	<0.001
CRP (mg/dL), Mean (SD)	7.0 (6.8)	6.9 (6.8)	7.4 (6.7)	0.27
Creatinine (mg/dL), Mean (SD)	1.10 (1.05)	1.11 (1.07)	1.09 (1.02)	0.82
Low eGFR (<60 mL/min), *n* (%)	345 (25.9)	247 (26.2)	98 (25.2)	0.73

Abbreviations: BMI, body mass index; WL, weight loss; NRI, Nutritional Risk Index; CRP, C-reactive protein; eGFR, estimated glomerular filtration rate. * For comparison between groups by Student’s *t*-Test (continuous variables) or Fisher’s exact test (categorical variables).

**Table 2:** Study population (*n* = 1373): Distribution by main admission diagnoses.

System/Group—Main Diagnosis	Total *n* (%)	*n* (%)
**Cardiovascular system**	104 (7.6)	
Ischemic heart disease		15 (1.1)
Heart failure		29 (2.1)
Peripheral arterious vascular diseases		12 (0.9)
Thromboembolic diseases		10 (0.7)
Arrhythmias		6 (0.4)
Valvular diseases (including infections)		9 (0.65)
Major surgery		18 (1.3)
Others (medical and surgical)		5 (0.35)
**Digestive system**	164 (11.9)	
Inflammatory bowel disease		49 (3.6)
Obstruction and diverticular disease		22 (1.6)
Acute biliary tract and pancreas illnesses		30 (2.2)
Chronic cirrhosis and/or hepatitis		25 (1.7)
Major abdominal surgery (others)		9 (0.65)
Other minor abdominal surgery		11 (0.8)
Infections		11 (0.8)
Other medical illnesses		7 (0.5)
**Malignancies**	640 (46.6)	
Head and neck		91 (6.6)
Gastrointestinal		168 (12.2)
Pancreas, liver and biliary tract		73 (5.3)
Lung and pleura		91 (6.6)
Hematology		102 (7.4)
Urinary		94 (6.8)
Other solid tumors		21 (0.4)
**Genito-urinary system**	56 (4.1)	
Renal failure		36 (2.6)
Infections		10 (0.7)
Kidney transplantation		4 (0.3)
Others (medical and surgical)		6 (0.4)
**Nervous system**	112 (8.2)	
Stroke		34 (2.5)
Dementia syndromes		25 (1.8)
Parkinson’s disease		14 (1.0)
Other neurodegenerative diseases		5 (0.35)
Infections		16 (1.2)
Hemorrhages		7 (0.5)
Others (medical & surgical)		11 (0.8)
**Orthopedics**	18 (1.3)	
Major surgery		10 (0.7)
Minor surgery		4 (0.3)
Medical		4 (0.3)
Respiratory system		115 (8.4)
Pneumonia		69 (5.0)
COPD		36 (2.6)
Lung transplantation		4 (0.3)
Other acute/chronic illnesses		6 (0.4)
**Infectious diseases**	70 (5.1)	
Sepsis		21 (1.5)
HIV		20 (1.45)
Tuberculosis		19 (1.4)
Other infections		10 (0.7)
**Miscellaneous**	94 (6.8)	
Dermatology		10 (0.7)
Endocrine and metabolic disorders		17 (1.2)
Other hematologic		9 (0.65)
Othorhinolaringology		9 (0.65)
Ophthalmology		2 (0.1)
Psychiatry		6 (0.4)
Rheumatology		25 (1.8)
Symptoms, signs and ill-defined conditions		16 (1.2)

Percentages are calculated according to full study population. Abbreviations: COPD, Chronic Obstructive Pulmonary Disease; HIV, Human Immunodeficiency Virus infection.

**Table 3:** Multivariate logistic regression of predictors of in-hospital mortality.

	*n*	OR (95% CI)	*p*-Value
**Male gender**	672	1.26 (0.89–1.77)	0.2
**Age**			0.69 *
18–64 years	540	1 (reference)	
65–79 years	527	1.17 (0.80–1.72)	0.41
≥80 years	306	1.01 (0.63–1.63)	0.96
**BMI category**			0.36 *
<18.5	397	1.31 (0.88–1.93)	0.18
18.5–24.9	709	1 (reference)	
25.0–29.9	215	0.84 (0.50–1.43)	0.53
≥30	52	0.70 (0.23–2.10)	0.53
**Six-month WL ≥ 10%**	623	1.53 (1.07–2.18)	0.019
**Surgical setting**	274	1 .72 (0.46–1.11)	0.14
**Cancer diagnosis**	640	1.40 (0.99–1.97)	0.054
**C-reactive protein**			0.002 *
<2.4 mg/dL	451	1 (reference)	
2.4–8.5 mg/dL	458	1.87 (1.16–3.02)	0.01
>8.5 mg/dL	464	2.45 (1.51–3.99)	<0.001
**eGFR < 60 mL/min**	345	1.88 (1.29–2.73)	0.001
**Prealbumin**			0.003 *
≥20 mg/dL	295	1 (reference)	
10–19 mg/dL	556	1.08 (0.63–1.85)	0.79
≤10μγ/δΛ	522	1.97 (1.15–3.37)	0.013
**Vitamin B12 > 1000 pg/mL**	396	2.20 (1.57–3.09)	<0.001

Abbreviations: OR, odds ratio; 95% CI, 95% confidence interval; BMI, body mass index; WL, weight loss; NRI, Nutritional Risk Index; CRP, C-reactive protein; eGFR, estimated glomerular filtration rate. * For trend over risk categories by multivariate analysis (*p*-values are reported in italics).

## Abbreviations

LOS	length of stay
NRI	nutritional risk index
BMI	body mass index
WL	weight loss
CRP	C-reactive protein
eGFR	estimated glomerular filtration rate
